# A Co-Culturing Approach Enables Discovery and Biosynthesis of a Bioactive Indole Alkaloid Metabolite

**DOI:** 10.3390/molecules25020256

**Published:** 2020-01-08

**Authors:** Fleurdeliz Maglangit, Qing Fang, Kwaku Kyeremeh, Jeremy M. Sternberg, Rainer Ebel, Hai Deng

**Affiliations:** 1 Marine Biodiscovery Centre, Department of Chemistry, University of Aberdeen, Meston Walk, Old Aberdeen, Aberdeen AB24 3UE, UK; r01qf16@abdn.ac.uk (Q.F.); r.ebel@abdn.ac.uk (R.E.); 2 Department of Biology and Environmental Science, College of Science, University of the Philippines Cebu, Lahug, Cebu City 6000, Philippines; 3 Marine and Plant Research Laboratory of Ghana, Department of Chemistry, University of Ghana, P.O. Box LG56 Legon-Accra, Ghana; kkyeremeh@ug.edu.gh; 4 Institute of Biological and Environmental Sciences, University of Aberdeen, Aberdeen AB24 3UE, UK; jsternberg@abdn.ac.uk

**Keywords:** silent genes, cryptic genes, co-culture, *Streptomyces* sp. MA37, *Pseudomonas* sp., indolocarbazole, alkaloid

## Abstract

Whole-genome sequence data of the genus *Streptomyces* have shown a far greater chemical diversity of metabolites than what have been discovered under typical laboratory fermentation conditions. In our previous natural product discovery efforts on *Streptomyces* sp. MA37, a bacterium isolated from the rhizosphere soil sample in Legon, Ghana, we discovered a handful of specialised metabolites from this talented strain. However, analysis of the draft genome of MA37 suggested that most of the encoded biosynthetic gene clusters (BGCs) remained cryptic or silent, and only a small fraction of BGCs for the production of specialised metabolites were expressed when cultured in our laboratory conditions. In order to induce the expression of the seemingly silent BGCs, we have carried out a co-culture experiment by growing the MA37 strain with the Gram-negative bacterium *Pseudomonas* sp. in a co-culture chamber that allows co-fermentation of two microorganisms with no direct contact but allows exchange of nutrients, metabolites, and other chemical cues. This co-culture approach led to the upregulation of several metabolites that were not previously observed in the monocultures of each strain. Moreover, the co-culture induced the expression of the cryptic indole alkaloid BGC in MA37 and led to the characterization of the known indolocarbazole alkaloid, BE-13793C **1**. Neither bacterium produced compound **1** when cultured alone. The structure of **1** was elucidated by Nuclear Magnetic Resonance (NMR), mass spectrometry analyses and comparison of experimental with literature data. A putative biosynthetic pathway of **1** was proposed. Furthermore, BE-13793C **1** showed strong anti-proliferative activity against HT-29 (ATCC HTB-38) cells but no toxic effect to normal lung (ATCC CCL-171) cells. To the best of our knowledge, this is the first report for the activity of **1** against HT-29. No significant antimicrobial and anti-trypanosomal activities for **1** were observed. This research provides a solid foundation for the fact that a co-culture approach paves the way for increasing the chemical diversity of strain MA37. Further characterization of other upregulated metabolites in this strain is currently ongoing in our laboratory.

## 1. Introduction

Gram-positive bacteria of the genus *Streptomyces,* are a prolific source of specialised metabolites. This property has led to the isolation and characterization of molecules that constitute more than half of the commercially available drugs, drug leads, and other bioactive compounds [1,2,3]. Whole-genome sequence analyses combined with recent advances in bioinformatics indicate that *Streptomyces* genomes contain many putative biosynthetic gene clusters (BGCs) that encode for bioactive metabolites [4,5,6,7,8,9]. However, a majority of these genes are silent or cryptic under normal laboratory culture conditions [10,11], and these “silent genes” represent a treasure trove of potentially promising novel metabolites with relevant biosynthetic pathways that require new approaches to induce their expression [7].

Recently, co-cultivation of *Streptomyces* with other microorganisms has received considerable interest for the discovery of cryptic natural products [7,12,13,14,15]. Bacteria do not live in isolation in the environment but rather in complex microbial communities interacting, sharing, and exchanging metabolic processes and signals [16]. These microbial interactions are often characterized by competition for limited resources or space availability and antagonism, which trigger the activation of silent gene clusters, leading to the production of bioactive specialised metabolites as defence mechanisms. Co-culturing represents an attempt to imitate this highly interactive setting in the laboratory, in which competition is deliberately provoked between two or more growing organisms in anticipation that cryptic BGCs are activated and transcribed under stressed co-culture conditions [7]. Co-cultures can be carried out on solid agar plates [17]; however, this Petri-dish method represents a drawback when large amounts of metabolites are required for isolation, bioassay, and *de novo* structure characterization [7]. Co-cultivation can also be performed in liquid substrates by growing two species in one culturing vessel, commonly referred to as mixed fermentation [18,19,20]. For example, the co-culture between the soil-dwelling bacterium *Streptomyces rapamycinicus* and the fungus *Aspergillus nidulans* induced the expression of a silent fungal gene cluster to yield the archetypal polyketide orsellinic acid and analogues [20]. The cryptic meroterpenoid pathway in the fungus *Aspergillus fumigatus* was expressed by co-cultivation with the same bacterium to produce the prenylated polyketides, fumicyclines [19]. 

**Figure 1:**
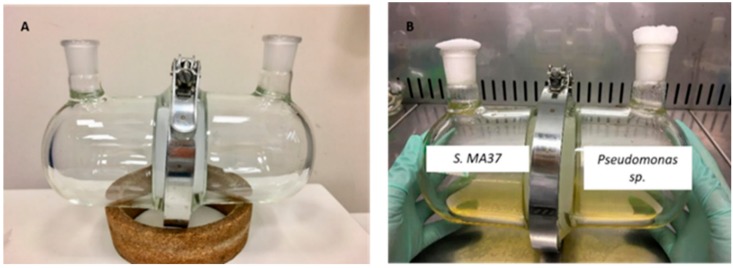
(**A**). Assembled co-culture device enabling separation of two independent cultures with a 0.22 μm membrane filter and joined by a holding clamp. (**B**). Co-culture of *Streptomyces* sp. MA37 and *Pseudomonas* sp. prior to incubation.

Continued screening for Gram-positive bacteria in our laboratory led to the isolation of a novel Ghanaian strain, *Streptomyces* sp. MA37. This strain is a prolific producer of natural products including legonmycins [21], neocarazostatin A [22,23,24,25], accramycin A [26], legonoxamines [27], and organofluorines [9,28]. However, genomic analysis of the MA37 strain suggested that only a small part of the biosynthetic genes encoding for specialised metabolites were expressed under laboratory culture conditions. In order to induce the expression of potentially valuable cryptic natural products encoded in the genome, we have carried out a co-culture fermentation by growing MA37 with the Gram-negative bacterium, *Pseudomonas* sp. Herein, we also present an experimental setup that allows co-culturing of two organisms while keeping them physically separated by a semi-permeable membrane but allowing exchange of nutrients and metabolic signals. Through this device (Figure 1), we can mimic the complex ecological interactions in their microhabitats while minimizing some variables present in cell–cell contact in the case of mixed fermentations.

**Figure 2:**
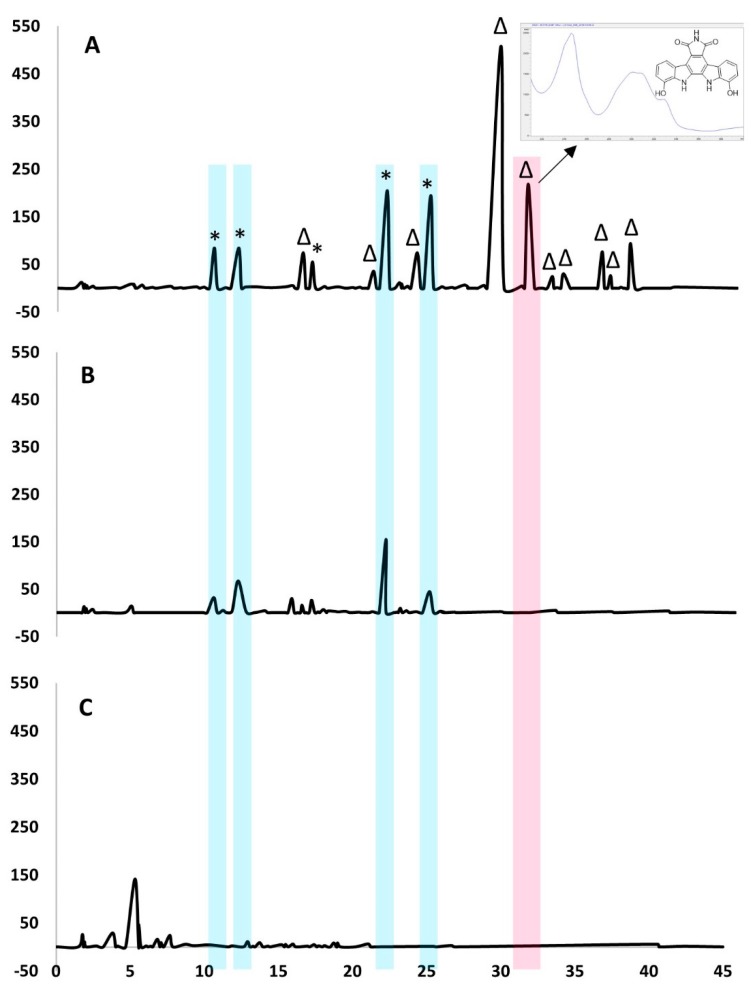
(**A**) HPLC traces recorded at λ250 nm of the extracts (10 µL injection, 15.0 mg/mL in methanol) of *Streptomyces* sp. MA37 cultivated with *Pseudomonas* sp., showing metabolites that are upregulated (*) and expressed only in co-cultures (**∆**). (**B**) *Streptomyces* sp. MA37 monoculture, and (**C**) *Pseudomonas* sp. monoculture.

The co-culture of strain MA37 and *Pseudomonas* sp. upregulated the production of many metabolites that were not previously detected in the bacterial monocultures (Figure 2), and the isolation of the known pyrroloindolocarbazole alkaloid metabolite, BE-13793C **1**. Neither bacterium produced **1** alone under our laboratory conditions. Hence, compound **1** is co-culture specific, and it is likely that the interactions between the two species induced the silent indolocarbazole gene cluster in MA37 to produce **1**. 

## 2. Results and Discussion

For the co-cultivation of the two strains, we developed a setup made of 2 modified glass vessels with a 100 mm opening on each compartment for sampling, inoculation, and filling purposes (Figure 1). The two chambers were joined by a holding clamp made of steel and an O-ring made of silicone to ensure a leak-proof sealing. A 0.22 µm polyvinylidene fluoride (PVDF) membrane filter (Sigma, UK) was used to separate the two culturing vessels. This setup allows two species to grow in separate chambers under identical culture conditions but with no direct contact. The semi-permeable membrane filter allows the diffusion of chemical nutrients and metabolic signals, thus ensuring microbial communication between two interacting species. The co-culture device was assembled and autoclaved prior to use. A similar setup was also previously described in the literature to co-culture two bacterial strains [12,29].

### 2.1. HPLC-UV Profiles of Co-Culture- and Monoculture-Derived Extracts from Strain MA37 and Pseudomonas sp.

Each compartment of our experimental setup containing 250 mL of ISP2 broth was inoculated with seed cultures of *Streptomyces* sp. MA37 and *Pseudomonas* sp., respectively. The 0.22 μm membrane filter allowed each strain to grow independently while in constant interaction/communication with another organism. The cultures were incubated for 7 days at 28 ℃ with constant agitation at 180 rpm to ensure a high metabolic exchange. Laboratory culture conditions for both bacterial species were maintained identical.

Toward the end of the fermentation, the growth in the chamber containing *Pseudomonas* sp. was observed to be inhibited and the culture became transparent. Subsequently, Diaion^®^ HP-20 (3 g/50 mL) was added to the culture, which was incubated for another 24 h and filtered under vacuum, after which the resin and mycelia were exhaustively extracted with 100% methanol. The methanol extract was concentrated under reduced pressure. The co-culture was repeated several times, ensuring identical culture conditions for every fermentation batch in order to obtain a total combined crude extract of about 9.0 g.

In addition, seed cultures of MA37 and *Pseudomonas* sp. were individually inoculated in 250 mL ISP2, as control cultures. They were both incubated at 28 °C, 180 rpm for 7 days, and the extraction was performed as described above.

Crude extracts of co- and mono-cultures (15.0 mg/mL in methanol) were screened by high-pressure liquid chromatography (HPLC)-ultraviolet (UV) analysis (Agilent Technologies 1260 Infinity, UK) with spectral scanning from 230–320 nm and a linear gradient from 0–100% methanol. Metabolite profiling by HPLC-UV and high-resolution electrospray ionization mass spectrometry (HRESIMS) demonstrated that several metabolites in the monocultures were upregulated in the co-cultured fermentations (Figure 2 designated as *). Moreover, metabolites that were not previously observed in the axenic cultures were detected in the co-culture (∆). Hence, these metabolites are co-culture specific and are likely produced by microbial interactions between *Pseudomonas* sp. and MA37 [30]. Several peaks were observed at λ250 nm in the co-culture, suggesting that they could be related compounds.

The co-culture crude extract was fractionated by vacuum liquid chromatography using a mixture of hexane–ethyl acetate–methanol gradient to yield 10 fractions (CC1-CC10). Most of the compounds that were not in the monocultures were present in fraction CC2; hence, further purification of this fraction was carried out by reversed-phase HPLC to yield a yellow powder, BE-13793C **1** (4.0 mg).

### 2.2. Structure Elucidation

**Figure 3:**
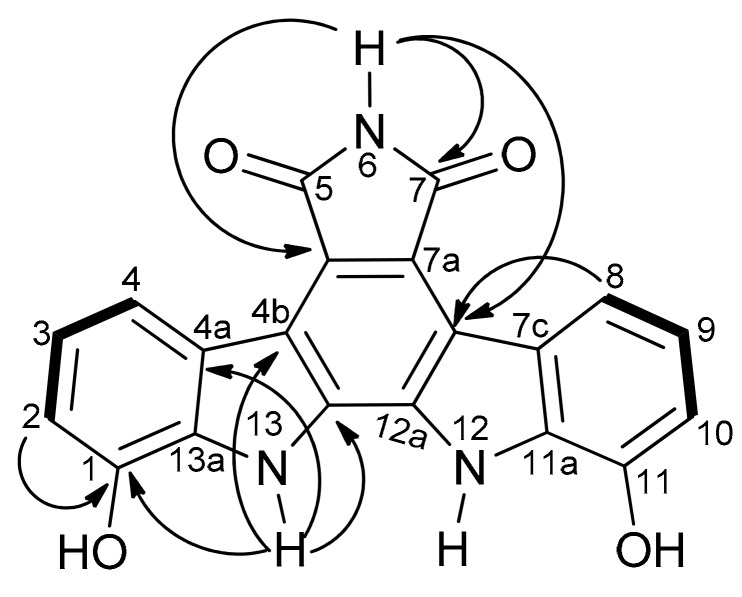
Structure of BE-13793C **1** with ^1^H-^1^H Correlation Spectroscopy (COSY) (**―**) and key Heteronuclear Multiple Bond Correlations (HMBC) (**→**).

The structure of **1** was characterized using spectrometric and spectroscopic analyses including liquid chromatography mass spectrometry (LCMS), 1D and 2D nuclear magnetic resonance spectroscopy (NMR), and also by comparison with literature data [31]. BE-13793C **1** (Figure 3) was obtained as a yellow powder with a molecular formula of C_20_H_11_N_3_O_4_ deduced by high-resolution electrospray ionization mass spectrometry (HRESIMS) (observed [M + H]^+^ = 358.0826, *m/z* calculated [M + H]^+^ = 358.0822; ∆ = 1.0576 ppm) with 17 degrees of unsaturation (Figure S1). The UV spectrum λmax 245, 305, 330, 395 nm suggested the presence of an indolocarbazole skeleton [32,33].

Analysis of the ^1^H-NMR spectrum in DMSO-*d*_6_ revealed that the integral ratio of the peak at δ_H_ 10.87 was half compared to the other signals (Figure S2). The ^1^H-NMR spectrum showed only 6 signals, 4 of which had an integration of 2. Three of these signals were attributed to the 6 aromatic methine protons at δ_H_ 6.98 (d, *J* = 8.2), δ_H_ 7.13 (tr), δ_H_ 8.41 (d, *J* = 7.8), and the remaining signals corresponded to the five exchangeable protons OH or NH (δ_H_ 10.63, 2H; δ_H_ 10.87, 1H; δ_H_ 11.57, 2H). The number of carbon atoms observed in HRESIMS were twice the number observed in the ^13^C-NMR data (Table 1). These results suggested that **1** had a symmetrical structure.

Inspection of the ^1^H-^1^H COSY correlations identified the aromatic spin systems H-2 to H-4 and H-8 to H-10, indicative of two 1,2,3-trisubstituted benzene rings (Figure S5). The HMBC correlations from NH-13 (δ_H_ 11.57) to C-1 (δ_C_ 143.3), C-13a (δ_C_ 130.7), C-12a (δ_C_ 129.0), C-12b (δ_C_ 129.0), C-4a (δ_C_ 123.0), C-4b (δ_C_ 115.9), and NH-12 (δ_H_ 11.57) to C-11 (δ_C_ 143.3), C-11a (δ_C_ 130.7), C-12a (δ_C_ 129.0), C-12b (δ_C_ 129.0), C-7c (δ_C_ 123.0), and C-7b (δ_C_ 115.9) supported the presence of an indolocarbazole moiety in the structure (Figure S4). The cross peaks from N-H (δ_H_ 10.87) to the carbonyl carbons at δ_C_ 171.7 (C5 and C7), and aromatic quaternary carbons at δ_C_ 120.3 (4c and 7a), and δ_C_ 115.9 (4b and 7b) established the connectivity of the pyrrole ring to the indolocarbazole framework.

The structure of **1** was confirmed to be the known compound BE-13793C **1**, the hydroxylated version of arcyriaflavin A [34] by comparison with the spectral data in the literature [31]. This indolocarbazole alkaloid was previously isolated from a *Streptoverticillium* strain and was found to inhibit topoisomerases I and II (IC_100_ 2 µM) and doxorubicin-resistant (IC_50_ 1.0 µM) or vincristine-resistant P388 murine leukemia cell lines (IC_50_ 0.7 µM). It caused in vivo inhibition of Erlich ascite tumour cells in mice [31,35]. Furthermore, **1** was also isolated from the mixed fermentation culture of mycolic-acid-containing bacteria, namely *Tsukamurella pulmonis* and *Streptomyces cinnamoneus* NBRC 13,823 [36].

### 2.3. Proposed Biosynthetic Pathway 

*In silico* analysis of the annotated genome of *Streptomyces* sp. MA37 using antiSMASH 3.0 [37] identified one putative *bec* biosynthetic gene cluster (BGC) likely to be involved in the production of BE-13793C **1**, showing high homology to rebeccamycin (AB071405.1) [32,38], staurosporine (AB088119.1) [39,40,41,42], K252a (FJ031030) [43], and AT2433 (DQ297453) [44] BGCs.

**Figure 4:**
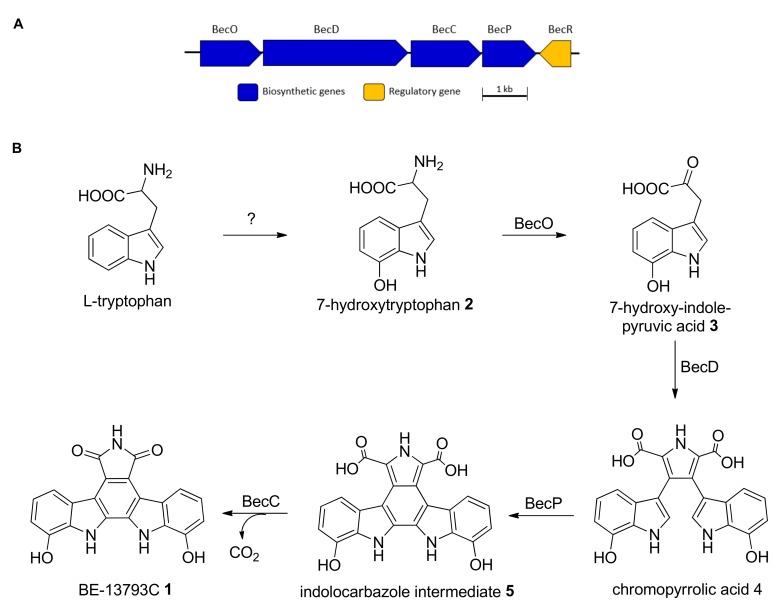
(**A**) Biosynthetic gene cluster of BE-13793C **1** in *Streptomyces* sp. MA37 and (**B**) Proposed biosynthetic pathway of **1**.

The *bec* BGC spans about 8.4 kb of genomic DNA containing open reading frames (ORFs) with deduced functions that were assigned based on database comparison and sequence analysis (Table 2, Figure 4A). Four ORFs (*becO, becD, becC, becP*) were found to have, on average, 50–60% identity to the corresponding ORFs of the staurosporine (*sta*), rebeccamycin (*reb*), k252a *(nok)*, and AT2433 (*atm*) clusters at the amino acid sequence level, hence we propose that these genes are involved in the indolocarbazole core formation (Figure S6, Table S1) [39,40,43].

Based on previous knowledge, the indolocarbazole core of rebeccamycin and staurosporine is derived from 2 units of l-tryptophan [32,39,45]. It was speculated that the biosynthesis of BE-13793C **1** begins from the hydroxylation of L-tryptophan to 7-hydroxytryptophan **2**; however, no gene that is encoded for tryptophan hydroxylase was identified within or in close proximity in the putative BGC of **1**. Further biochemical characterization is required to identify the enzyme involved in the conversion. 

The oxidative deamination of two molecules of 7-hydroxy-tryptophan **2** to the corresponding indole-3-pyruvic acid **3** is catalysed by the putative enzyme BecO [32,42,46,47]. Supplementation of DL-fluorotryptophans to cultures of *Saccharothrix aerocolonigenes* induced the production of novel fluoro-indolocarbazole rebeccamycin derivatives [48], suggesting that *rebO* could accept tryptophan analogues for oxidative deamination. Likewise, we propose that BecO with 56% amino acid identity to RebO performs a similar function.

Gene expression elucidated that **3** is solely required by the tetrameric hemoprotein StaD or RebD for the production of the chromopyrrolic acid **4** in staurosporine or rebeccamycin biosynthesis [45,49], contrary to the previous reports that both **2** and **3** are the precursors for the coupling reaction to yield **4** [38,45]. BecD encoded for 1095 amino acid residues. A BLAST (Basic Local Alignment Search Tool) search indicated that it is closely related to StaD (60% amino acids identity) and RebD (54% amino acids identity) and is likely to involve a similar coupling reaction of two molecules of **3** and NH_4_^+^ in BE-13793C biosynthesis in MA37 for the formation of **4**. Sequential oxidative steps occur through the action of additional putative enzymes, including BecP (cytochrome P450 oxygenase) and BecC (Flavin Adenine Dinucleotide (FAD)-binding monooxygenase) to convert **4** to the dicarboxylic bisindole **5,** then to the indolocarbazole skeleton **6**. A database search suggested that BecR is similar to the luxR family transcriptional regulator and shares 31% and 44% similarity to RebR and StaR, respectively. It is speculated that BecR could function as a transcriptional activator of the expression of BE-13793C biosynthetic genes in MA37. [38]. Gene-disruption experiments showed no production of rebeccamycin-related compounds in the *rebR*-truncated mutants. However, it is vital for production in the heterologous expression of indolocarbazole biosynthetic genes [18].

One of the structural differences between **1** and rebeccamycin/staurosporine is the presence of the sugar moiety. Detailed analysis of the annotated MA37 genome showed no rebG, StaG, atmG, or NokL homologues that encode for the N-glycosyltransferase [38,42,43,44]. The RebH and RebF halogenase enzymes in rebeccamycin BGC were also not found in the *bec* cluster of MA37. This explains the production of only the aglycone and deschloro-indolocarbazole in the coculture-induced fermentation of MA37 and *Pseudomonas* sp.

### 2.4. Biological Activity Test

Several natural and synthetic indolocarbazole alkaloids such as staurosporine [39,50,51,52,53], arcyriaflavins [34,54], ED-110 [35], rebeccamycin [32,55], and related analogues have been widely studied for their antibacterial [39,56], anti-tubercular [39,57], antifungal [56], anticancer [35,58,59], cytotoxic activities [60], as well as inhibition against human African trypanosomiasis (HAT) [52,61]. In fact, a number of these analogues, UCN-01 [62], BMY-27557 [63], XL119 [64], NSC 655,649 [39,40], have already entered clinical trials. Considering this, we examined the antimicrobial, anticancer, cytotoxic activities, as well as activity against trypanosomes, of the isolated alkaloid, BE-13793C **1**.

### 2.5. Antimicrobial/Antibiofilm Assays for BE-13793C ***1***

BE-13793C **1** was tested against a panel of Gram-positive (Enterococcus faecalis, Staphylococcus aureus, methicillin-resistant Staphylococcus aureus, Streptococcus B., Staphylococcus haemolyticus) and Gram-negative pathogens (Escherichia coli, Pseudomonas aeruginosa) (Table 3); however, it did not show any significant activity at the highest concentration tested (140 µM).

The biofilm-forming bacteria, *Staphylococcus epidermidis* was used to evaluate the antibiofilm activity of **1**. The activity was determined by quantification of the total biofilm biomass with crystal violet; however, it did not display inhibition of biofilm formation at concentrations between 0–140 µM.

### 2.6. Antiproliferative/Cytotoxicity Activities for BE-13793C ***1***

BE-13793C **1** showed strong antiproliferative activity against human HT-29 colorectal adenocarcinoma (ATCC HTB-38) cells (IC_50_ 3.16 µM). The cytotoxicity of **1** was also investigated on normal lung (ATCC CCL-171) cells by colorimetric MTS (3-(4,5-dimethylthiazol-2-yl)-5-(3-carboxymethoxyphenyl)-2-(4-sulfophenyl)-2*H*-tetrazolium) assay. There was no significant difference in cell viability between the test compound and the media control at 0–140 µM, indicating that **1** inhibited cell proliferation, induced anoikis, and caused cell death of HT-29 colon cells but had no cytotoxic effect on normal cells (Table 4, Figure S7). Several biological activities have been reported for indolocarbazole alkaloids including antituberculosis [65], anticancer [55], and antitumour [31,55] activities. However, there are no records in the literature for the bioactivity of BE-13793C **1** in HT-29 cell-based bioassays. The standard antibiotic, staurosporine (Sigma, UK), which differed from **1** in the presence of the sugar unit, displayed a potent antiproliferative activity (IC_50_ 0.20 µM) consistent with the reported antiproliferative properties of staurosporine in the literature [50,66]. Midostaurin (sold under the name Rydapt^®^), a semi-synthetic analogue of staurosporine, had been approved for clinical use in 2017 for the treatment of acute myeloid leukemia (AML) [50].

### 2.7. Trypanosome Inhibition Assay for BE-13793C ***1***

The protozoan parasite *Trypanosoma brucei* spp. causes trypanosomiasis in humans and livestock [67] and was used as a model for antiprotozoal activity in **1**. BE-13793C **1** was tested against *T. brucei brucei* following the protocols described by Raz [68] (1997) and Pimentel-Elardo [52] (2010), with minor modifications. The antibiotic standard, staurosporine (Sigma), which primarily differs with **1** in the presence of the sugar unit in the structure, showed potent inhibition against *T. brucei brucei* (IC_50_ = 0.022 µM) similar to the reported activity in literature [52]. However, BE-13793C **1** did not show any growth inhibition against the trypanosomes at the highest concentration tested (50 µM) (Table 5). The antiparasitic activity of staurosporine is attributed to its potent inhibition of protein kinase C (PKC) [50,69,70]. The sugar unit attached to two indole nitrogens in staurosporine plays a key role in its activity against trypanosomatid parasites [71]. Previous studies have shown that the aglycone counterpart does not show any significant PKC inhibition [55,72], which explains the inactivity of **1** against *T. brucei brucei*. Reviews on protein kinases as drug targets in trypanosomes are described in the literature [70,73].

## 3. Materials and Methods 

### 3.1. General Experimental Procedures

The infrared spectrum was obtained on a PerkinElmer Spectrum version 10.4.00 Fourier transform infrared (FTIR) spectrometer (2013) (Scotland, UK) equipped with an attenuated total reflection (ATR) diamond cell. High-resolution electrospray ionisation mass spectrometry (HR-ESIMS) data were obtained using an LTQ Orbitrap Thermo Scientific MS system coupled to a Thermo Instrument HPLC system (Accela PDA detector, Accela PDA autosampler, and Accela pump). The following conditions were used: capillary voltage of 45 V, capillary temperature of 200 °C, auxiliary gas flow rate of 10−20 arbitrary units, sheath gas flow rate of 25−40 arbitrary units, spray voltage of 4.5 kV, and mass range of 100−2000 amu (maximal resolution of 30,000). For HRESIMS, a C18 Sunfire analytical HPLC column (5 μm, 4.6 mm × 150 mm) was used with a mobile phase consisting of 5−100% acetonitrile with 0.1% formic acid over 30 min at a flow rate of 0.5 mL/min. Reversed-phase HPLC separations were performed using a C18 column (5 μm, 100 Å, 10 mm × 250 mm) connected to a binary pump and monitored using a photodiode array detector. NMR data were acquired on high-performance digital Bruker AVANCE III HD 400 MHz (Ascend™ 9.4 Tesla, UK) and Bruker AVANCE III HD 600 MHz (Ascend™ 14.1 Tesla, UK) with Prodigy TCI™ cryoprobe at 25 °C.

### 3.2. The Autoclavable Co-Culture Experimental Setup

The co-culture setup consisted of two modified flasks (each with a capacity of 600 mL) with a 100 mm flat edge opening and a 30 mm neck (Figure 1) [74]. Each chamber was joined by a holding clamp and an O-ring made of silicone to ensure a leak-proof sealing. A 0.22 µm polyvinylidene fluoride (PVDF) membrane filter (Durapore^®^, Ireland) was used to separate the two culturing vessels. Each flask had a 30 mm opening for filling and sampling purposes, which was covered by foam stoppers (Fisherbrand^TM^ polyurethane) during culturing. The culture vessels, O-ring, clamp, and membrane filter were assembled and autoclaved prior to use.

### 3.3. Strains, Genomic DNA, and Media

The *Streptomyces* sp. MA37 strain was isolated from the soil sample collected from Legon-Accra, Ghana Africa [9]. The *Pseudomonas* sp. was isolated from the rhizosphere of a conker tree (*Aesculus hippocastanum*) growing along Meston Walk, University of Aberdeen, Old Aberdeen, Scotland, UK. The genomic DNA of the latter was extracted and was identified by 16S gene RNA sequencing (MN720504) [75]. All media/broth for fermentation were obtained from Fisher Scientific (UK) unless otherwise stated.

### 3.4. Fermentation and Extraction

The seed cultures of *Streptomyces* sp. MA37 and *Pseudomonas* sp. were prepared by inoculating the bacterial glycerol stocks in ISP2 broth (yeast extract 4 g, malt extract 10 g, glucose 4 g, in 1 L H2O, 1:1000) and incubating for 5 days at 28 °C with shaking at 180 rpm (Incu-shake FL16-2).

Each co-culture partition vessel was filled with up with 250 mL of ISP2 and inoculated with the seed cultures of MA37 in one chamber and *Pseudomonas* sp. in the other. The co-culture was incubated for 7 days in the rotary shaker maintained at 28 °C and 180 rpm. Subsequently, Diaion^®^ HP-20 resin (3 g/50 mL) was added to the culture and incubated for 24 h at the same temperature and shaking conditions. The resin was filtered, washed with H_2_O, extracted with MeOH (3 × 500 mL), and concentrated under reduced pressure (Buchi Rotavapor R200, Scotland, UK). The extracts were subjected to LCMS analysis. The co-culture fermentation was repeated several times (approx. 10 L ISP2) to obtain 9g of the total crude extract.

Monocultures of *Streptomyces* sp. MA37 and *Pseudomonas* sp. were cultured in ISP2 for 7 days at 28 °C and 180 rpm in conical flasks (Pyrex™ borosilicate glass narrow neck flasks) and extracted with HP-20 resin as described above.

### 3.5. Chromatography and Isolation

Crude extracts of co- and mono-cultures (5.0 mg/mL in methanol) were initially screened by HPLC-UV analysis (Agilent Technologies 1260 Infinity) with spectral scanning from 230−320 nm and a linear gradient from 0–100% methanol.

The co-culture crude was then fractionated by vacuum liquid chromatography on silica gel 60 (Acros Organics™ ultra-pure 60A 40-63u), eluting with a gradient of n-hexane−ethyl acetate−MeOH to give 10 subfractions (CC1-CC10). HPLC-UV-HRESIMS analysis revealed that most metabolites that were not observed in the monocultures were in CC2; hence, further purification of this fraction was carried out by reversed-phase HPLC (Agilent Technologies 1260 Infinity) (ACE C18-HL 10 µM 10 × 250 mm) equipped with a diode array detector (DAD). HPLC separation was performed using solvent A (95% H_2_O, 5% methanol, and 0.1% trifluoroacetic acid) and solvent B (100% methanol) as eluents, with a linear gradient from 20%−100% MeOH over 40 min and a solvent flow rate of 1.5 mL min^−1^. All of the employed chemicals were HPLC-grade. The separation afforded 4.0 mg of an indolocarbazole alkaloid, BE-13793C **1**.

BE-13793C: yellow solid. UV (PDA) λmax: 245, 305, 330, 395nm; IR (neat) ν_max_ (cm^−1^): 3427, 1740, 1590, 1422, 1343, 1205, 857 nm; ^1^H, ^13^C-NMR data, see Table 1; HR ESIMS (positive mode) *m/z* calculated [M + H]^+^ = 358.0822; observed [M + H]^+^ = 358.0826; ∆ = 1.0576 ppm.

### 3.6. AntiBacterial Assay

All strains used for the antibacterial tests were from the American Type Culture Collection (ATCC): *Escherichia coli* (25922), *Pseudomonas aeruginosa* (27853), *Staphylococcus aureus* (25923), Methicillin-resistant *S. aureus* (33591), *Enterococcus faecalis* (29212), *Streptococcus* B (12386), and *Staphylococcus haemolyticus* (clinical isolate 8-7A). Gentamicin (Sigma) was used as the standard antibiotic. The antibacterial assay of **1** was performed as described in our previous report [26].

### 3.7. Antibiofilm Assay

The antibiofilm activity of **1** was determined by quantification of the total biofilm biomass with crystal violet. The biofilm-forming bacteria *Staphylococcus epidermidis* were inoculated in 96-well plates with clear bottoms (Corning, NY, USA) containing the Mueller Hinton (MH) medium (with 1% glucose to induce biofilm) and the test compound (140 μM). The plate was incubated overnight at 37 °C. Wells were carefully washed with 100 μL phosphate-buffered saline (PBS) to eliminate free-floating bacteria. Biofilm formation was fixed by incubating the plates at 65 °C for 1 h followed by staining with 0.1% crystal violet (100 μL) for 10 min. Excess stain was thoroughly rinsed off with distilled water, and plates were left to dry at 65 °C for at least 1 h. The optical density (600 nm), OD_600_, of stained biofilm was measured on a Victor3 Plate Reader. OD_600_ is a measure of the bacteria adhering to the surface and forming biofilms, and readings below 0.25 were considered active. *S. epidermidis* and milliQ water comprised the positive control. *Staphylococcus haemolyticus*, which does not form biofilms, was used as the negative control. Experiments were performed in triplicate.

### 3.8. Antiproliferative/Cytotoxicity Assays

The antiproliferative activity of **1** was tested on the human HT29 colon carcinoma (ATCC HTB-38). Cell lines (2000 cells/well) were added to 96-well plates (Nunc, Thermo Fisher Scientific, USA) in Dulbecco’s Modified Eagle Medium (DMEM) containing 10% foetal bovine serum (FBS) and gentamicin (10 µg/mL). Cells were incubated for 24 h at 37 °C and maintained in a humidified atmosphere of 5% CO2 and low passage. The lung normal cell lines (ATCC CCL-171) were used for toxicity testing of **1**. Cell lines (4000 cells/well) were added to 96-well plates and incubated in similar conditions as described above.

At the end of 24 h, the test compound (0.1, 1, 2.5, 5, 10, 12.5, 25, and 50 µg/mL) was added to the wells and incubated for 72 h. Cell viability was determined by a colorimetric MTS assay using a tetrazolium dye, 3-(4,5-dimethylthiazol-2-yl)-5-(3-carboxymethoxyphenyl)-2-(4-sulfophenyl)-2*H*-tetrazolium, inner salt, and an electron coupling reagent, phenazine methosulfate (PMS). Cell Titer 96^®^ Aqueous One Solution Reagent (Promega, Madison, WI, USA) (10 µL) was added to each well and then incubated for 1 h at 37 ^°^C. The absorbance was recorded on a plate reader at 490 nm. The number of living cells was determined by measuring their ability to reduce the tetrazolium salt to the coloured formazan product at 490 nm. Staurosporine (Sigma) was used as standard reference for the antiproliferative and cytotoxity assays. Assays were performed in triplicate.

### 3.9. Trypanosome Inhibition Assay

The trypanosome inhibition assay was performed using the Alamar Blue assay as described by Raz [68] and Pimentel-Elardo [52], with minor modifications. Bloodstream form *Trypanosoma brucei brucei* cells (Isolate STIB247, [76]) were cultured in Hirumi’s Modified Iscove’s (HMI-9) medium with the modifications that foetal bovine serum (FBS) was increased to 15% (*v/v*) and serum Plus^TM^ was omitted [77].

A defined number of parasites, 1 × 10^5^ trypanosomes mL^−1^ (5 µL), were seeded in each well (Corning™ clear polystyrene 96-well sterile microplate) containing HMI-9 medium and the test compound (previously dissolved in 2% sterile DMSO) (100 µL). Alamar blue (resazurin sodium salt, 0.05 mg/mL, Sigma) was added to the wells. Assays were performed in triplicate and incubated for 20−23 hrs at 37 °C in an atmosphere of 5.0% CO_2_. Staurosporine (Sigma) was used as the reference antibiotic. The positive control consisted of the trypanosomes in culture medium with 2% DMSO, while the negative control was comprised of the medium without the trypanosomes. The absorbance was measured on a SpectraMax ABS Plus microplate reader (Molecular Devices, Wokingham, UK) at a wavelength of 570 nm and reference wavelength of 600 nm.

## 4. Conclusions 

The co-culture of *Streptomyces* sp. MA37 and *Pseudomonas* sp. allowed the production of a pyrroloindolocarbazole alkaloid, BE-13793C **1.** Compound **1** was not observed in the bacterial monocultures; thus, it is co-culture specific. It is likely that the interactions between the two bacterial species induced the silent indolocarbazole gene cluster in *Streptomyces* sp. MA37 to produce **1**. BE-13793C **1** showed strong antiproliferative activity against HT29 (ATCC HTB-38) cells at IC_50_ 3.16 µM but no toxic effect on normal lung (ATCC CCL-171) cells. This is the first report for the activity of **1** against HT-29. Compound **1** was tested for its antimicrobial and anti-trypanosomal activities, but it did not show any significant activity at 0−140 µM. A putative biosynthetic pathway of BE-13793C **1** was proposed. The current research also provides more evidence of the ability of co-culturing to alter the chemical diversity derived from any given microbial strain, as indicated in the current very popular “one strain many compounds” (OSMAC) philosophy.

## Figures and Tables

**Table 1:** ^1^H and ^13^C-NMR of BE-13793C **1** at 600 MHz and 298 K.

No.	^1^H			^13^C ^a^	
	α (ppm)	mult.	J (Hz)	α (ppm)	Type
1	10.63 (OH)	s		143.3 ^b^	C
2	6.98	d	8.2	111.4	CH
3	7.13	tr	8.2	121.3	CH
4	8.41	d	7.8	115.5	CH
4a	-	-	-	123.0	C
4b	-	-	-	115.9	C
4c	-	-	-	120.3	C
5	-	-	-	171.7	C
6	10.87 (NH)	s		-	-
7	-	-	-	171.7	C
7a	-	-	-	120.3	C
7b	-	-	-	115.9	C
7c	-	-	-	123.0	C
8	8.41	d	8.2	115.5	CH
9	7.13	tr	8.2	121.3	CH
10	6.98	d	7.8	111.4	CH
11	10.63(OH)	s	-	143.3 ^b^	C
11a	-	-	-	130.7	C
12	11.57 (NH)	s	-	-	-
12a	-	-	-	129.7	C
12b	-	-	-	129.7	C
13	11.57 (NH)	s	-	-	-
13a	-	-	-	130.6	C

^a^ Obtained from Heteronuclear Single Quantum Correlation (HSQC) and Heteronuclear Multiple Bond Correlation (HMBC) in CD_3_OD and DMSO-*d*_6_, respectively; ^b^ estimated from DMSO-*d*_6_ spectrum.

**Table 2:** Deduced functions of open reading frames (ORFs) in BE-13793C (*bec)* in the *Streptomyces* sp. MA37 biosynthetic gene cluster.

Gene	Deduced Function	AA
*becO*	amino acid oxidase	508
*becD*	chromopyrrolic acid synthase	1095
*becC*	Flavin-binding monooxygenase	540
*becP*	cytochrome P450 oxygenase	417
*becR*	LuxR response regulator	221

**Table 3:** Antimicrobial/antibiofilm tests against a panel of bacterial pathogens.

Pathogen	Minimum Inhibitory Concentration (MIC) (µM)	
	BE-13793C 1	Gentamicin
*Enterococcus faecalis* ATCC 29,212	>140	1.05
*Staphylococcus aureus* ATCC 25,923	>140	0.25
Methicillin-resistant *S. aureus*, MRSA ATCC 33591	>140	2.00
*Streptococcus* B. ATCC 12,386	>140	1.04
*Staphylococcus haemolyticus* clinical isolate 8-7A	>140	40.2
*Escherichia coli* ATCC 25,922	>140	0.25
*Pseudomonas aeruginosa* ATCC 27,853	>140	1.04
Biofilm *Staphylococcus epidermidis* ATCC 35,984	>140	17.8

**Table 4:** Antiproliferative/cytotoxic activities of **1**.

	Human Colon Carcinoma (HT29 ATCC HTB-38)	Normal Lung Cell ATCC CCL-171
BE-13793C **1** (IC_50_, µM)	3.16	>140
Staurosporine (IC_50_, µM)	0.20	>100

**Table 5:** Antitrypanosomal activity of **1**.

	IC_50_ (µM)
BE-13793C **1**	>50
Staurosporine	0.022

## Supplementary Materials

The Supplementary Materials are available online.
