# Supplementary material for: A Co-Culturing Approach Enables Discovery and Biosynthesis of a Bioactive Indole Alkaloid Metabolite
Source: Molecules. 2020 Jan 8;25(2):256. doi: 10.3390/molecules25020256 (PMC7024260; doi:10.3390/molecules25020256)
Supplement: Supplementary file 1 [file molecules-25-00256-s001.pdf]

SUPPLEMENTARY INFORMATION

A co-culturing approach enables discovery and biosynthesis of a bioactive indole alkaloid metabolite

Fleurdeliz Maglangit [1,2] *, Qing Fang [1], Kwaku Kyeremeh [3], Jeremy Sternberg [4], Rainer Ebel [1], Hai Deng [1] *

Contents Page

Figure S1: HR ESIMS of BE-13793C **1** 2

Figure S2: H-NMR of BE-13793C **1** (298K, 600MHz) 3

Figure S3: HSQC of BE-13793C **1** (298K, 600MHz) 4

Figure S4: HMBC of BE-13793C **1** (298K, 600MHz) 4

Figure S5: COSY of BE-13793C **1** (298K, 600MHz) 5

Table S1. Deduced functions of the ORFs in BE-13793C **1** BGC 6

Figure S6: Biosynthetic gene cluster comparison 6

Figure S7: MIC curve of BE-13793C **1** against HT29 (ATCC HTB-38) colon cells 7

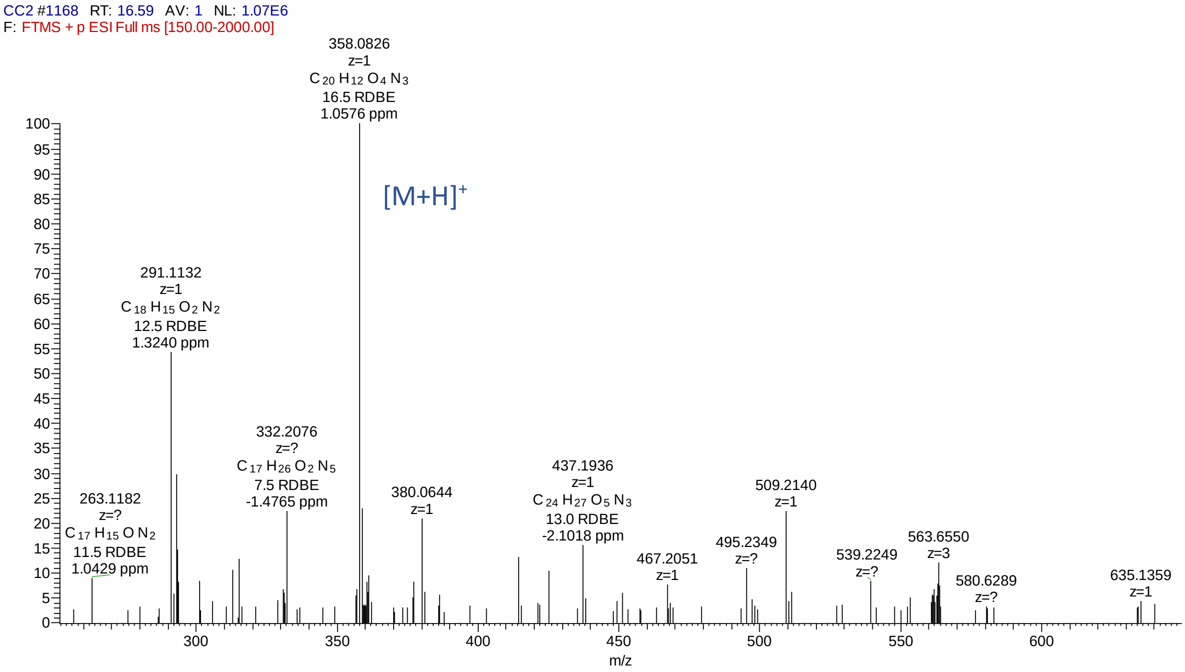

Figure S1. HR ESIMS of BE-13793C **1**

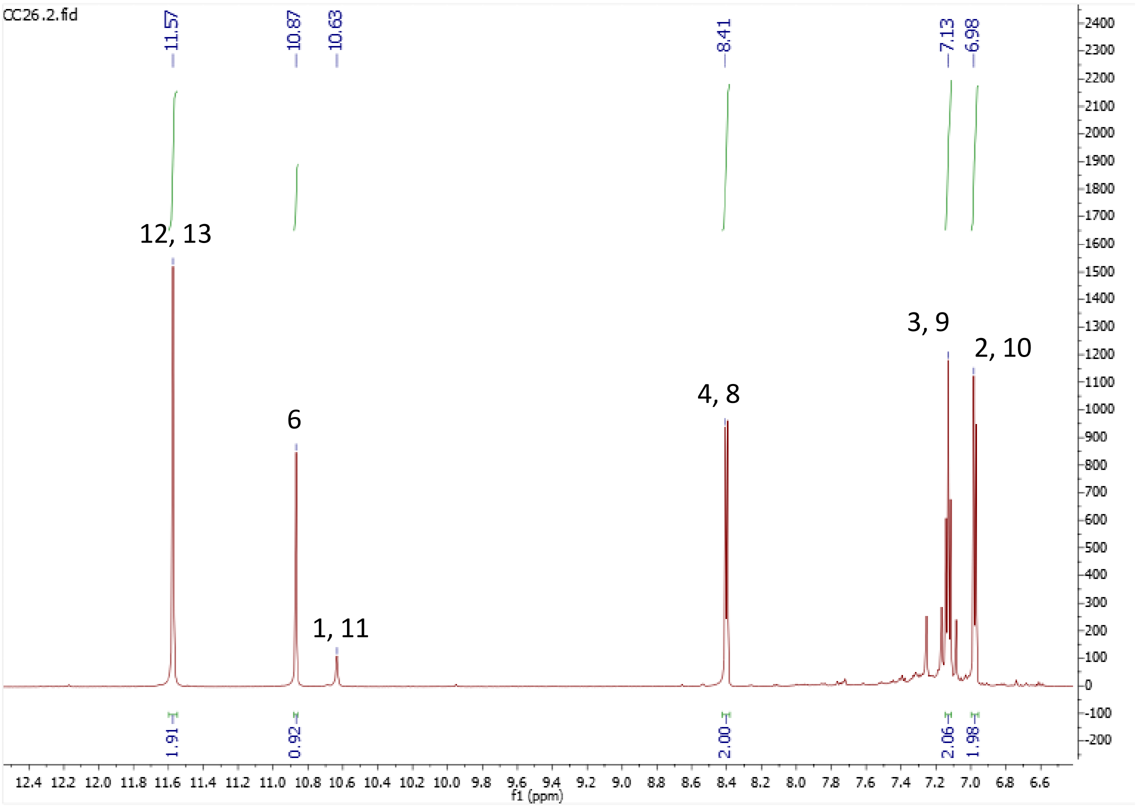

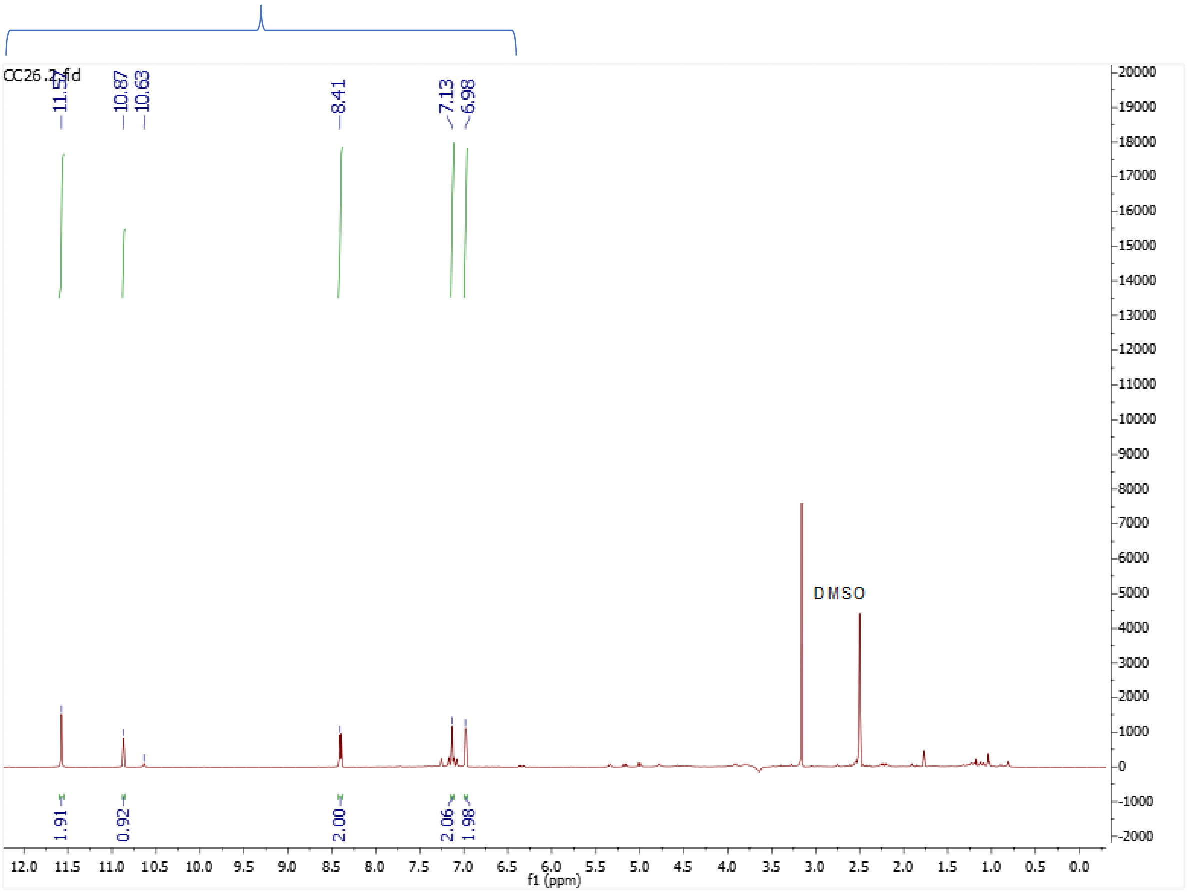

Figure S2: H-NMR of BE-13793C **1** (DMSO-d_6, 298K, 600MHz)

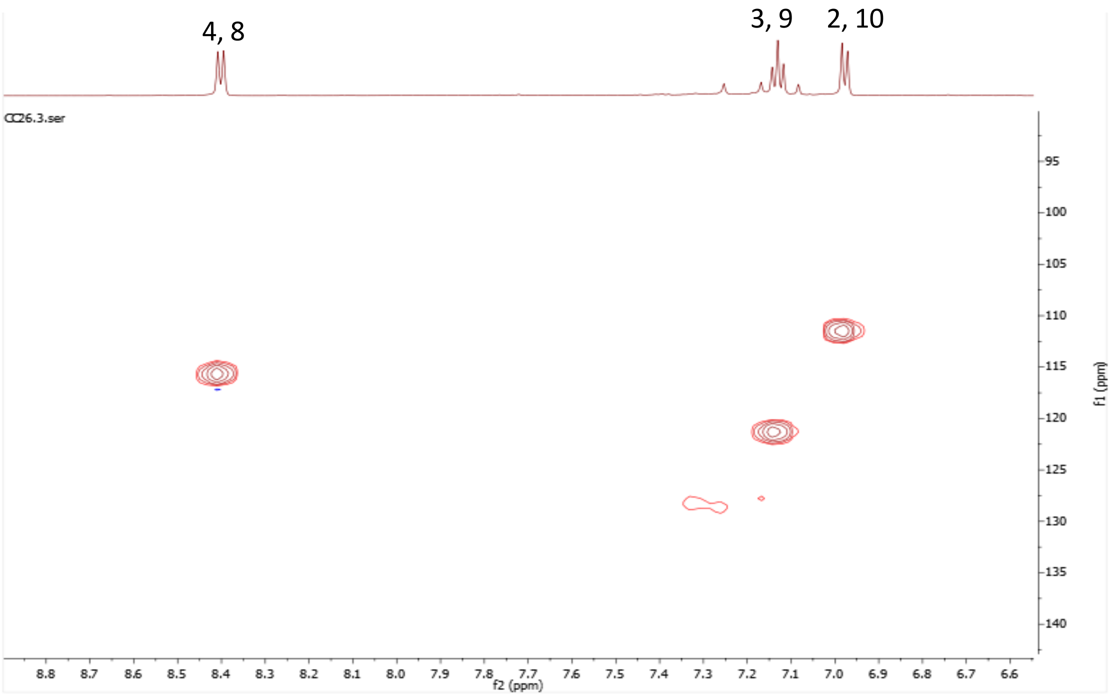

Figure S3: HSQC of BE-13793C **1** (CD_3OD, 298K, 600MHz)

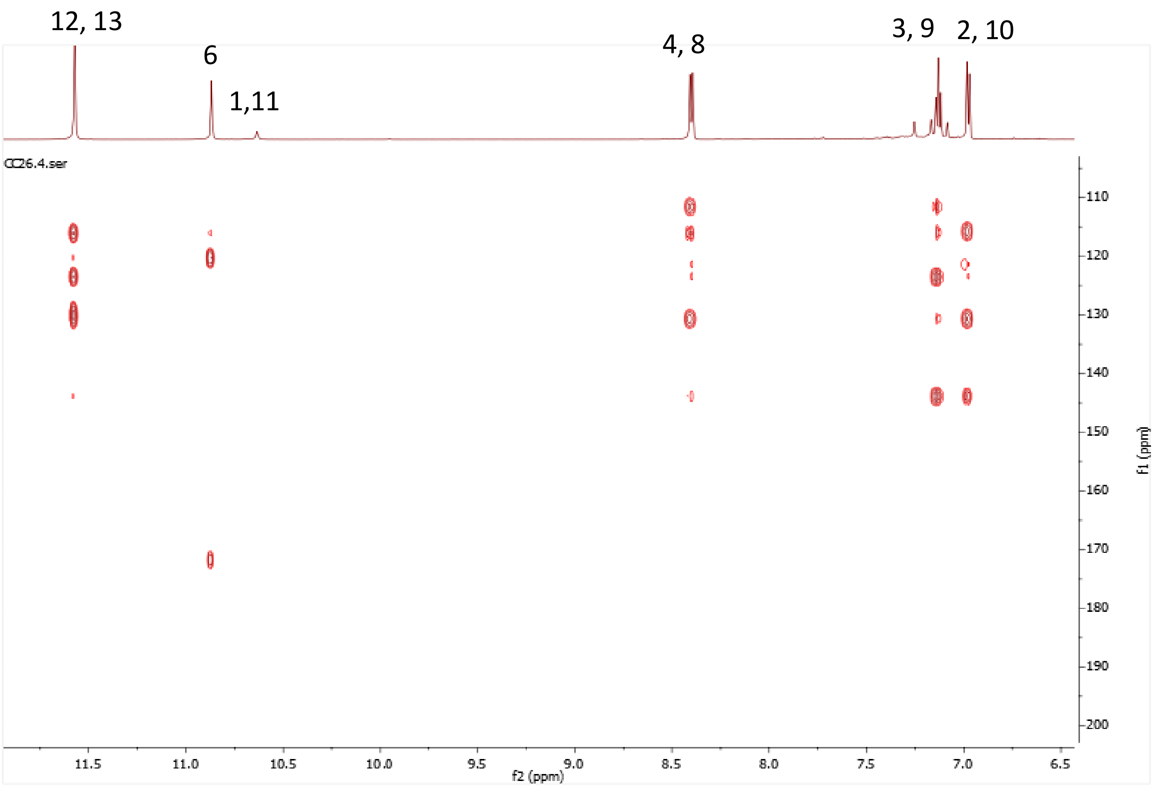

Figure S4: HMBC of BE-13793C **1** (DMSO-*d*$_6$, 298K, 600MHz)

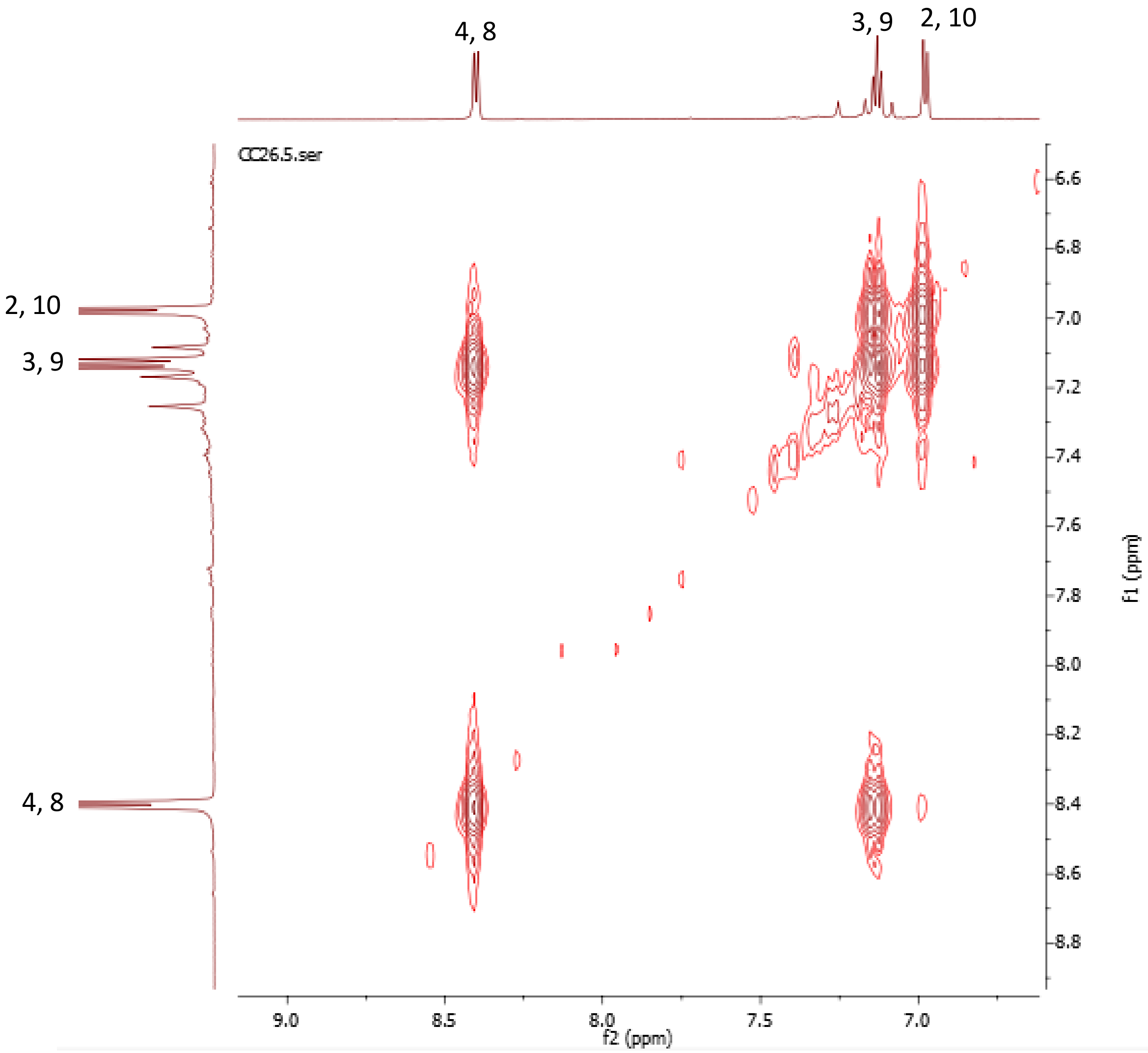

Figure S5: COSY of BE-13793C **1** (CD_3OD, 298K, 600MHz)

Table S1. Deduced functions of the ORFs in BE-13793C **1** (*bec*) biosynthetic gene cluster in *Streptomyces* sp. MA37 showing homology to the rebeccamycin (*reb*) BGC in *Lechevalieria aerocolonigenes* and staurosporine (*sta*) BGC in *Streptomyces* sp. TP-A0274

Gene	Deduced Function	AA	Staurosporine % Protein identity	Rebeccamycin % Protein identity
becO	FAD-dependent oxidoreductase	508	53%	56%
becD	bis-indole formation	1095	60%	54%
becC	FAD-binding monooxygenase	540	62%	61%
becP	cytochrome P450	417	51%	55%
becR	LuxR family DNA-binding response regulator	221	44%	31%

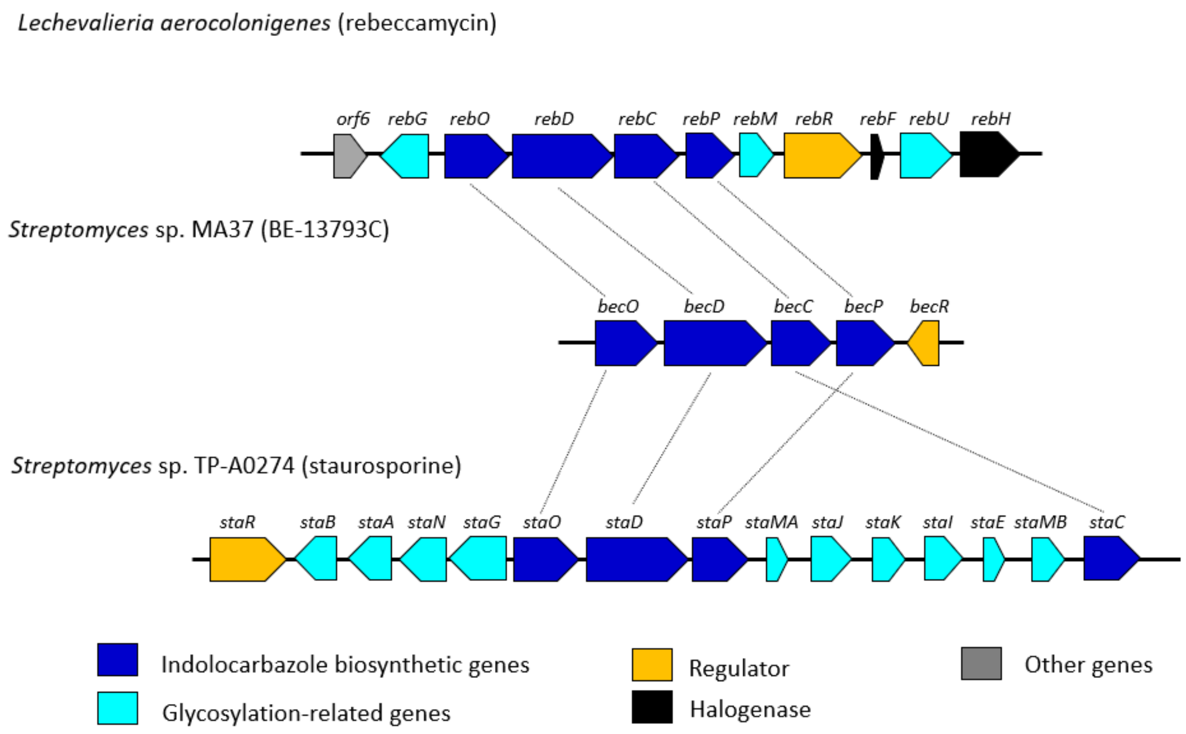

Figure S6: Biosynthetic gene cluster comparison of BE-13793C **1** in *Streptomyces* sp. MA37, rebeccamycin in *Lechevalieria aerocolonigenes,* and staurosporine in *Streptomyces* sp. TP-A0274

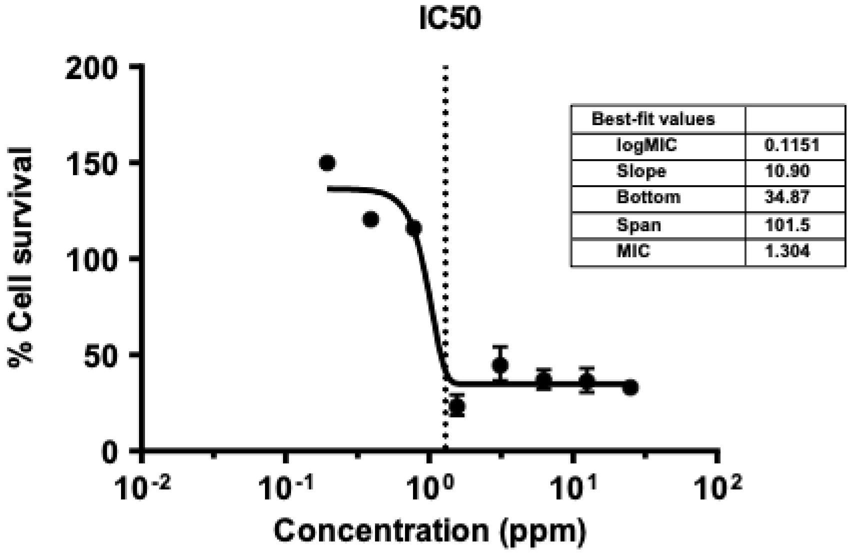

Figure S7. MIC curve of BE-13793C **1** against HT29 (ATCC HTB-38) colon cells
